# The overall and domain-specific quality of life of Chinese community-dwelling older adults: the role of intrinsic capacity and disease burden

**DOI:** 10.3389/fpsyg.2023.1190800

**Published:** 2023-08-25

**Authors:** Xiuhua Hu, Jian Ruan, Weibin Zhang, Jie Chen, Zhijun Bao, Qingwei Ruan, Zhuowei Yu

**Affiliations:** ^1^Laboratory of Aging, Anti-aging and Cognitive Performance, Shanghai Institute of Geriatrics and Gerontology, Huadong Hospital, Shanghai Medical College, Fudan University, Shanghai, China; ^2^Department of Otolaryngology, Huadong Hospital, Shanghai Medical College, Fudan University, Shanghai, China; ^3^Shanghai Key Laboratory of Clinical Geriatrics, Huadong Hospital, and Research Center of Aging and Medicine, Huadong Hospital, Shanghai Medical College, Fudan University, Shanghai, China; ^4^Department of Geriatrics, Huadong Hospital, Fudan University, Shanghai, China

**Keywords:** intrinsic capacity, multicomorbidity, sensory impairment, locomotion impairment, vitality, interpersonal relationships, demoralization, health-related quality of life

## Abstract

**Objective:**

This study aimed to investigate the impact of the different domains of intrinsic capacity (IC) and chronic disease burden on health-related quality of life (HRQoL) and domain-specific HRQoL in Chinese community-dwelling older adults.

**Design:**

A cross-sectional observational study of a community-based cohort.

**Participants:**

We evaluated Chinese older adults (*n* = 429, mean age, 72.91 ± 7.014 years; female proportion, 57.30%).

**Measurements:**

IC contains five domains, namely locomotion, vitality, cognition, psychological, and sensory capacity. Locomotion dysfunction was defined as grip and/or gait decline. Vitality decline was defined if two of the following three parameters were present: fatigue, physical inactivity, and weight loss or overweight. Cognition was classified into normal cognition, pre-mild cognitive impairment (pre-MCI), and MCI according to the normative *z*-scores of the neuropsychological test battery. Psychological dysfunction was diagnosed based on depressive symptoms. Sensory dysfunction was defined as hearing and/or vision impairment. HRQoL was assessed using the AQoL-8D scale, which comprised physical (including independent living, senses, and pain) and psychosocial (including mental health, happiness, self-worth, coping, and relationships) dimensions. Low HRQoL (HRQoL score or subscores in the highest quintile) was used as a dependent variable in logistic regression analyses adjusted for demographic, health-related, and psychological confounders.

**Results:**

Sensory impairment was an independent determinant of senses, and locomotion impairment was significantly associated with overall HRQoL, independent living, and pain in the physical dimension of HRQoL. Cognition was an independent determinant of the senses. Vitality was independently associated with overall HRQoL, senses, and pain in the physical dimension and mental health and relationships in the psychological dimension of HRQoL. The psychological domain of IC was independently associated with overall and domain-specific HRQoL apart from senses after adjustment for all confounders. The number of multimorbidities mainly had a significant impact on independent living after adjustment for all confounders.

**Conclusion:**

IC domains and chronic disease burden had heterogeneous influences on overall and domain-specific HRQoL. The impairment of sensory and locomotion domains had a synergistic impact on the overall and physical dimensions of HRQoL. The vitality and psychological domains of IC had more profound effects on HRQoL. Older people with high morbidity might have a higher risk of poor independent living.

## 1. Introduction

Healthy aging is the process of developing and maintaining functional ability and wellbeing in old age, regardless of the presence or absence of diseases (World Health Organization, 2015, 2017a). Survival in old age, delay in the onset of age-dependent diseases, and maintenance of optimal functional ability for the maximum period are the critical characteristics of healthy aging. To maintain or recover functional ability as suggested by the World Health Organization (WHO), integrated care for older people (ICOPE) based on intrinsic capacity (IC) framework was constructed to facilitate the shift from traditionally disease-centered clinical practice to function-centered clinical practice that extends an individual's health span without dependence (World Health Organization, 2019). Functional ability depends on the dynamic interplay between IC reserves and the external environment (i.e., physical and social determinants).

Cesari et al. (2018) identified that IC is mainly composed of five domains or dimensions: locomotion (including muscular function), vitality (including homeostatic regulation or energy metabolic capacity, and balance between energy intake and energy utilization), cognition, psychological (including mood and sociality), and sensory (including vision and hearing). IC reserves, which maintain the effective capacity reserves of an individual to undertake the physical and mental tasks of everyday life, are multiple, dynamic, and decline with age (World Health Organization, 2017b). The decline of IC reserves increases the vulnerability to homeostasis maintenance and environmental challenges throughout life. It also leads to adverse health outcomes such as disability, falls, handicaps, disease burden, hospitalization, and mortality (Clegg et al., 2013). The WHO construct of IC longitudinally predicts the decline in an individual's functional ability after adjusting for the number of multimorbidities and other confounders (Beard et al., 2021).

Recent studies have shown that IC, the environment, and disease burden could affect HRQoL. It has been reported that functional ability, defined as the interaction of neighborhood environments and IC, has a stronger impact on the HRQoL in older people than only IC (Stephens et al., 2020). Furthermore, IC was measured using the number of multimorbidities (Stephens et al., 2020). When the Integrated Care for Older People (ICOPE) screening tool for IC recommended by the WHO was used, individuals with a decline in IC were more likely to be older, frail, and disabled after being adjusted for age, sex, and multimorbidity. A higher IC was associated with better physical, mental, and total quality of life (QoL; Ma et al., 2020).

The different domains of IC also affect HRQoL. Subjective cognitive decline in middle-aged and older adults could have a negative impact on their mental health (Bouldin et al., 2021). The psychological domains of IC referred to as depressive symptoms, were significantly and directly associated with HRQoL and mediated the relationships between cognitive complaints and HRQoL (Toyoshima et al., 2021). Sensory and locomotion domains are not only particularly important dimensions of physical function but also critical components of both IC and frailty (Belloni and Cesari, 2019; Ma, 2019). Studies indicated that individuals with hearing and/or visual impairments have a significant association with lower QoL (Tseng et al., 2018; Harithasan et al., 2020; Zhang et al., 2021). The overall HRQoL and physical/mental component of HRQoL in individuals with dual sensory impairment were worse than that of those with only hearing or visual impairment (Ding et al., 2023). Mobility had a strong association with QoL (Henchoz et al., 2017) and could predict the change in HRQoL in older adults (Davis et al., 2015). An increase in physical activity and mobility could improve HRQoL in older adults (Eisele et al., 2015). In this study, we hypothesized that IC domains, especially sensory and locomotion domains, have different effects on the overall and domain-specific quality of life.

Multimorbidity is highly prevalent in the older population and also results in a decrease in HRQoL. The declining risk in the mental and physical dimensions of HRQoL was significantly associated with the increase in the number of multimorbidities (Makovski et al., 2019). Moreover, multimorbidity could interact with frailty and vision impairment and cumulatively affect the quality of life and key domains of IC (Ho et al., 2020). Multimorbidity in combination with the IC domains, such as cognitive impairment, significantly increases the loss of quality-adjusted life expectancy in middle-aged and older adults (Xiong et al., 2021). Therefore, it is necessary to compare the different effects of multimorbidity (chronic disease burden) and IC domains on overall and domain-specific HRQoL.

The purpose of this study was to explore the relationship between IC domains and HRQoL. The primary objective was to investigate the different effects of the impairment of IC domains, especially sensory and locomotion, on overall and domain-specific HRQoL. The second objective was to examine the effects of chronic disease burden on overall and domain-specific quality of life.

## 2. Materials and methods

### 2.1. Study design

**Figure 1:**
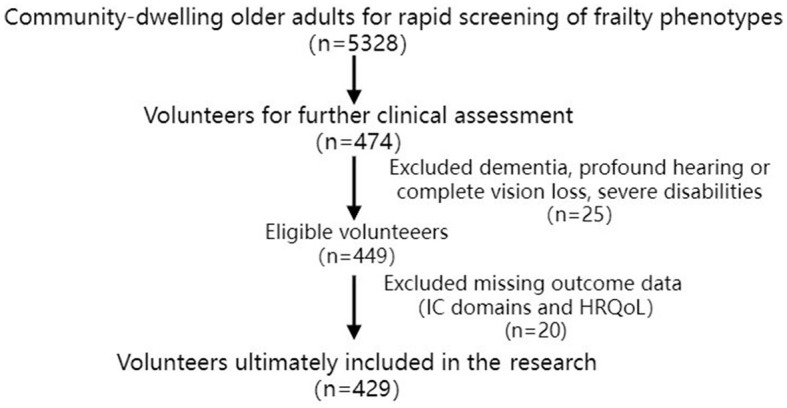
A flow diagram outlining the recruitment of community-dwelling older adults for the assessment of IC and HRQoL.

The participants were recruited through a population-based cross-sectional study of health promotion in older adults with frailty (Ruan et al., 2020). The cohort with 429 individuals was the same as that in our previous study concerning the effect of frailty phenotypes on age-related hearing loss (ARHL) and tinnitus (Ruan et al., 2021a), and the different effects of physical frailty and ARHL along with tinnitus on HRQoL (Zhang et al., 2021; Figure 1). Several variables, including nutritional status, sleep quality, and occupation, were excluded from the analysis due to >30% missing data. Missing values for the remaining variables were < 3%, and individuals with missing values were removed automatically from the cohort for statistical analysis.

### 2.2. Participants

All the participants underwent IC, HRQoL, and comprehensive geriatric assessments. The inclusion and exclusion criteria were reported in detail in our previously published article (Zhang et al., 2021). This study was approved by the ethics committee of Huadong Hospital (Approval No. Ref 2018K055) and was conducted in accordance with the Declaration of Helsinki. All participants provided written informed consent.

### 2.3. IC screening

Based on the definition of the five dimensions of IC, locomotion decline was determined based on if the individual had a slow gait and/or weak grip with Chinese reference values (Hao et al., 2017). In detail, a slow gait was diagnosed when walking time for 4.57 m was ≥7 s with a body height of ≤173 cm for males and ≤159 cm for females or ≥6 s with a body height of >173 cm for males and >159 cm for females. A weak handgrip for males was diagnosed when a handgrip of ≤29 kg with a body mass index (BMI) of ≤24.0 kg/m^2^, ≤30 kg with a BMI between 24.1 and 28.0 kg/m^2^, and ≤32 kg with a BMI of >28 kg/m^2^; for females was diagnosed when a handgrip of ≤17 kg with a body mass index (BMI) of ≤23 kg/m^2^, ≤17.3 kg with a BMI between 23.1 and 26.0 kg/m^2^, ≤18 kg with a BMI between 26.1 and 29.0 kg/m^2^, and ≤21 kg with a BMI of >29.0 kg/m^2^. Sensory impairment of IC was diagnosed when the individual was with hearing and/or visual impairment. Hearing impairment was assessed according to the pure-tone average (PTA) based on 0.5-, 1-, 2-, and 4-kHz thresholds using pure-tone audiometry. Individuals with a PTA of ≤25 dB experiencing tinnitus in the better ear were defined as having a hearing impairment. Vision referred to the participant's eyesight with glasses or corrective lenses if they were used regularly. Visual impairment was measured using a self-reported question asking participants to rate each sense on a 5-item Likert scale as excellent, very good, good, fair, or poor (Liljas et al., 2020). Only those reporting fair or poor were considered to have a visual impairment. Cognitive performance stratification was assessed using the normative *z*-scores of the neuropsychological test battery, which includes executive or attention, language, and memory domains (Ruan et al., 2021b). Psychological dimension decline, referred to as depressive symptoms, was assessed using the 15-item short form of the Geriatric Depression Scale (GDS; Zhang et al., 2020). The cutoff value of GDS ≥5 was referred as to having depressive symptoms. Fatigue, low physical activity, and abnormal nutrition could reflect homeostatic regulation or energy metabolic capacity and balance between energy intake and energy utilization (Schrack et al., 2010; Cesari et al., 2018; Bautmans et al., 2022). According to the assessment tool reported in the literature (Cigolle et al., 2007; Ma et al., 2020; Zhu et al., 2021), vitality decline was assessed using three parameters: fatigue, low physical activity, and abnormal weight according to the body mass index (BMI), which included unexplained weight loss or gain. Participants were classified as fatigued if an individual has 2–3 points to either of the two following questions about the somatic symptoms of The Center for Epidemiologic Studies-Depression Scale (Callahan and Wolinsky, 1994): (1) I felt that everything I did was an effort and (2) I could not get “going.” (0 point: <1 d; 1 point: 1–2 d; 2 points: 3–4 d; 3 points: >4 d). Individuals were defined as having a low physical activity if total kilocalories expended on leisure-time physical activity of <383 kcal/w for males and <270 kcal for females (approximately the same with 2 h walking; Taylor et al., 1978). The unexplained weight loss or gain was defined as Chinese reference values (BMI <18.5 or ≥24 Kg/m^2^; Zeng et al., 2014). Vitality decline was defined based on if an individual had more than two of the three aforementioned parameters.

### 2.4. Chronic multimorbidity and other covariates

The chronic burden was referred to as the number of chronic multimorbidities, which were categorized as 0, 1, 2, and >2. A total of 13 chronic multimorbidities reported in our previous studies (Ruan et al., 2020, 2021a,b) were included: diabetes mellitus, cardiovascular disease (CVD, including myocardial infarction/heart attack or angina pectoris; hypertension, congestive heart failure, or cardiac arrhythmia), osteoporosis, stroke, arthritis, chronic obstructive lung disease, anemia, peripheral vascular disease, Alzheimer's disease, Parkinson's disease, mental or psychiatric disorders, chronic renal disease, and non-skin malignancy. Other covariates include age, sex, education level, self-reported smoking, and alcohol intake.

### 2.5. HRQoL assessment

The 35-item Assessment of Quality of Life-8-Dimension (AQoL-8D) self-report questionnaire, which contains three physical (independent living, senses, and pain) and five psychosocial (mental health, happiness, self-worth, coping, and relationships) dimensions (Richardson et al., 2014), was used for the assessment of HRQoL. The standardized total HRQoL score and subscore of each specific domain in both physical and psychosocial dimensions ranged from 0 to 100, with lower scores indicating higher HRQoL. As reported in our previous study, the highest quintile for the HRQoL score and each domain-specific HRQoL subscore was defined as 1 = low HRQoL, while scores below the highest quintile were defined as 0 = better HRQoL (Zhang et al., 2021). In the current study people, we verified that age-related hearing loss with tinnitus and physical frailty had different effects on the overall and domain-specific quality of life assessed by using this questionnaire (Zhang et al., 2021).

### 2.6. Statistical analysis

The null hypothesis (H0) of the study was that there is no effect of IC domains on overall and domain-specific cognitive performance. Accordingly, the alternative hypothesis (Ha) was that there is a significant effect of IC domains on overall and domain-specific cognitive performance. The alpha level (*p* ≤ 0.05, two sides) was set to reject H0 and accept Ha. The continuous variables were expressed as medians and quartiles. A bivariate correlation was analyzed using Pearson's test for normally distributed variables and Spearman's test for non-normal distribution variables and *X*^2^ for categorical variables. The difference among the four groups based on sensory and locomotion impairment for continuous variables was tested using univariate ANOVA analysis. When the homogeneity of variances was inappropriate, the Mann–Whitney *U*-test was employed to analyze the univariate correlation. The association between the different domains of IC or the number of multimorbidities with low overall HRQoL scores, and each low subscore of domain-specific HRQoL was further assessed using multiple logistic regression analyses adjusted for age, sex, and education levels (Model 1), adjusted for covariates in Model 1 and other health covariates, including smoking, drinking, multimorbidities, vitality, and cognitive performance (Model 2), and adjusted for covariates in Model 2, and GDS scores (Model 3). A *P*-value of < 0.05 was considered statistically significant, and all statistical analyses were performed using the SPSS software (version 18.0).

## 3. Results

### 3.1. Participant characteristics

The participants were stratified according to whether they had sensory or locomotion impairment of IC. The demographic, medical, and psychological characteristics are shown in Supplementary Table 1. Among lack of sensory and locomotion, sensory impairment, locomotion impairment, and sensory and locomotion groups, the bivariate analysis indicated a significant difference in parameters, including age, smoking, drinking, multimorbidity, vitality, and cognitive performance based on the normative *z*-score of the neuropsychological test battery.

### 3.2. The influences of sensory and locomotion impairment in HRQoL and domain-specific HRQoL

The distribution of participants with higher total HRQoL scores, which means lower HRQoL, or higher domain-specific HRQoL subscores, which means lower domain-specific HRQoL, among four groups (lack of sensory and locomotion impairment, sensory impairment, locomotion impairment, and sensory and locomotion impairment groups) is indicated in Table 1. The average standardized scores of HRQoL and subscores of domain-specific HRQoL are indicated in Table 2. The multiple logistic regression analysis of the association of sensory and/or locomotion impairment with HRQoL indicated that sensory and/or locomotion impairment significantly affects overall HRQoL and the physical, but not psychosocial dimension of HRQoL (Table 3). Individuals with less severe locomotion impairment [odds ratio (OR) = 5.285; 95% confidence interval (CI) = 2.310–12.093] and sensory impairment (OR = 2.442; 95% CI = 1.316–4.533) significantly increased the odds of higher HRQoL or lower total scores of HRQoL than those with both sensory and locomotion impairments (Model 1). However, the significant influence disappeared after it was adjusted for additional health-related (Model 2) and additional psychological (Model 3) confounders.

**Table 1:** Distribution of low HRQoL (high scores) and domain-specific HRQoL (0 = low HRQoL scores or domain-specific subscores, 1 = high HRQoL scores or domain-specific subscores).

	**Total sample**	**Lack of sensory and locomotion impairment**	**Sensory impairment**	**Locomotion impairment**	**Sensory and locomotion impairment**	***X*^2^-value**	***p* value**
**Total HRQoL**	426					19.055	0.000
0	350	25	165	13	147		
1	76	15	43	2	16		
**Physical dimension**							
Independent living	423					20.301	0.000
0	328	22	152	12	142		
1	95	17	54	3	21		
Senses	423						
0	241	7	124	3	107	38.465	0.000
1	182	32	82	12	56		
Pain	423						
0	267	20	118	10	119	12.289	0.006
1	156	19	88	5	44		
**Psychological dimension**							
Mental health	423					1.783	0.619
0	343	29	166	12	136		
1	80	10	40	3	27		
Happiness	423					6.201	0.102
0	317	26	147	12	132		
1	106	13	59	3	31		
Self-worth	423					2.005	0.571
0	370	32	183	14	141		
1	53	7	23	1	22		
Coping	423					7.195	0.66
0	378	33	178	15	152		
1	45	6	28	0	11		
Relationships	424					7.211	0.065
0	343	27	164	13	139		
1	81	13	42	2	24		

**Table 2:** The average normative scores of HRQoL and subscores of domain-specific HRQoL in different study groups.

	**Lack of sensory impairment**		**Sensory impairment**		**Mobility impairment**		**Sensory and mobility impairment**		***F*-value**	***p***
	**Average value** ±**standard deviation**	**95% CI**	**Average value** ±**standard deviation**	**95% CI**	**Average value** ±**standard deviation**	**95% CI**	**Average value** ±**standard deviation**	**95% CI**		
**Total HRQoL**	82.713 ± 12.533	78.704–86.721	77.649 ± 12.976	75.876–79.423	78.392 ± 8.262	73.817–82.968	72.201 ± 12.228	70.310–74.093	10.216	0.000
**Physical dimension**										
Independent living	87.749 ± 9.381	84.708–90.790	81.392 ± 14.038	79.463–83.320	78.519 ± 16.781	69.226–87.812	72.290 ± 16.972	69.665–74.916	16.782	0.000
Senses	80.671 ± 10.421	77.293–84.049	69.081 ± 12.848	67.316–70.846	81.539 ± 8.625	76.762–86.315	67.060 ± 14.005	64.894–69.226	15.796	0.000
Pain	80.769 ± 20.051	74.270–87.269	78.155 ± 20.036	75.403–80.908	75.333 ± 18.074	65.324–85.342	68.650 ± 22.839	65.118–72.183	7.382	0.000
**Psychological dimension**										
Mental health	81.896 ± 12.131	77.963–85.828	77.449 ± 14.430	75.467–79.432	81.212 ± 11.859	74.645–87.779	75.014 ± 14.525	72.767–77.261	3.117	0.026
Happiness	77.564 ± 17.719	71.820–83.308	73.786 ± 17.657	71.361–76.212	68.750 ± 14.752	60.580–76.920	68.022 ± 18.535	65.155–70.888	4.747	0.003
Self-worth	76.068 ± 32.454	65.548–86.589	77.427 ± 17.232	75.060–79.794	73.333 ± 14.840	65.115–81.552	72.495 ± 19.425	69.490–75.449	1.941	0.122
Coping	83.547 ± 11.705	79.753–87.341	78.843 ± 15.456	76.720–80.966	81.111 ± 12.781	74.033–88.189	72.853 ± 16.371	70.321–75.385	7.589	0.000
Relationships	80.556 ± 15.161	75.707–85.404	74.991 ± 14.155	73.047–76.935	78.765 ± 9.014	73.773–83.757	71.302 ± 14.162	69.112–73.493	5.732	0.001

**Table 3:** Multiple logistic regression analysis of the association of sensory and/or locomotion impairment with high quality of life (low score) and low subscores in domain-specific quality of life.

	**Low quality of life score**			**Independent living**			**Senses**			**Pain**		
**Stratification of sensory and/or mobility (ref: 3)**	**Model 1 OR (95% CI)**	**Model 2 OR (95% CI)**	**Model 3 OR (95% CI)**	**Model 1 OR (95% CI)**	**Model 2 OR (95% CI)**	**Model 3 OR (95% CI)**	**Model 1 OR (95% CI)**	**Model 2 OR (95% CI)**	**Model 3 OR (95% CI)**	**Model 1 OR (95% CI)**	**Model 2 OR (95% CI)**	**Model 3 OR (95% CI)**
Lack of sensory and locomotion impairment	5.285 (2.310, 12.093)^***^	-	-	5.225 (2.392, 11.414)^***^	3.458 (1.487, 8.039)^**^	4.338 (1.786, 10.533)^***^	6.915 (2.819, 16.962)^***^	3.175 (1.230, 8.194)^*^	3.490 (1.273, 9.571)^*^	2.569 (1.255, 5.262)^**^	-	-
Sensory impairment	2.442 (1.316, 4.533)^**^	-	-	2.418 (1.390, 4.207)^**^	1.631 (0.902, 2.949)	**1.737 (0.934, 3.231)**	1.072 (0.686, 1.674)	0.724 (0.441, 1.188)	0.799 (0.482, 1.323)	2.034 (1.306, 3.167)^**^	-	-
Locomotion impairment	1.310 (0.266, 6.452)	-	-	1.844 (0.475, 7.159)	1.455 (0.357, 5.934)	0.335 (0.039, 2.851)	7.592 (2.012, 28.646)^**^	5.393 (1.387, 20.976)^*^	4.581 (1.129, 18.586)^*^	1.503 (0.477, 4.729)	-	-

OR, odds ratios; CI, confidence intervals; GDS, the Geriatric Depression Scale.

Model 1 is adjusted for age, gender, education, and social dysfunction. Model 2 is adjusted for covariates in model 1 and health-related factors, including self-reported smoking, alcohol intake, chronic comorbidities, and cognition based on normative z-scores of neuropsychological test battery. Model 3 is adjusted for covariates in model 2 and GDS. Ref 3 = sensory and locomotion impairments.

* p < 0.05;

** p < 0.01; and

*** p < 0.001; bold values denote marginally statistical significance.

Among the three physical domains, compared to those with sensory and locomotion impairment, individuals with a lack of sensory and locomotion impairment had better independent living and senses after being adjusted for confounders in Model 1 (OR = 5.225, 95% CI = 2.392–11.414; OR = 6.915, 95% CI = 2.819–16.962), Model 2 (OR = 3.458, 95% CI = 1.487–8.039; OR = 3.175, 95% CI = 1.230–8.194), and Model 3 (OR = 4.338, 95% CI = 1.786–10.533; OR = 3.490, 95% CI = 1.273–9.571), respectively, and they also experienced less pain (OR = 2.569, 95% CI = 1.255–5.262) in Model 1. Individuals with sensory impairment had better independent living in Model 1 (OR = 2.418, 95% CI = 1.390–4.207) and Model 3 (OR = 1.737, 95% CI = 0.934–3.231) and experienced less pain in Model 1 (OR = 2.034, 95% CI = 1.306–3.167). Individuals with locomotion impairment had better senses after being adjusted for confounders in Model 1 (OR = 7.592, 95% CI = 2.012–28.646), Model 2 (OR = 5.393, 95% CI = 1.387–20.976), and Model 3 (OR = 4.581, 95% CI = 1.129–18.586).

### 3.3. The influences of other domains of IC and chronic disease burden in overall HRQoL and physical dimension of HRQoL

Apart from older age, low education level significantly increased the odds of adverse influence in the senses (OR = 0.950, 95% CI = 0.917–0.983) and pain (OR = 0.897, 95% CI = 0.837–0.961) domains of HRQoL after being adjusted for confounders in Model 3. Multiple logistic regression analysis of the association of other domains of IC, and chronic burden with HRQoL indicated that the cognitive impairment, vitality, and psychological domains of IC, and chronic burden had different influences on HRQoL and the physical dimension of HRQoL (Table 4). Individuals with high vitality had a significantly high likelihood of better HRQoL in Model 2 (OR = 7.791, 95% CI = 2.377–25.529) and Model 3 (OR = 4.824, 95% CI = 1.434–16.231), better independent living in Model 1 (OR = 2.345, 95% CI = 1.044–5.268), better senses in Model 2 (OR = 3.106, 95% CI = 1.751–5.512) and Model 3 (OR = 2.965, 95% CI = 1.658–5.301), and less pain in Model 2 (OR = 2.720, 95% CI = 1.496–4.948) and Model 3 (OR = 2.455, 95% CI = 1.278–4.718). Compared to those with MCI, individuals with normal cognition (OR = 2.339, 95% CI = 1.314–4.165; OR = 2.357, 95% CI = 1.302–4.268) and with pre-MCI (OR = 1.941, 95% CI = 1.023–3.681; OR = 1.915, 95% CI = 0.997–3.678) had a significantly higher likelihood of better senses in Models 2 and 3, respectively. The psychological domain of IC significantly affected HRQoL and independent living and the pain domains of the physical dimension of HRQoL. Those with low capacity of IC psychological domain (depressive symptoms) had a significantly lower likelihood of better HRQoL (OR = 0.754, 95% CI = 0.649–0.876), better independent living (OR = 0.839, 95% CI = 0.746–0.943), and less pain (OR = 0.823, 95% CI = 0.741–0.914) in Model 3.

**Table 4:** Multiple logistic regression analysis of the association of the decline of other IC domains with high quality of life (low score) and low subscores in domain-specific quality of life.

	**Low quality of life score**			**Independent living**			**Senses**			**Pain**		
	**Model 1 OR (95% CI)**	**Model 2 OR (95% CI)**	**Model 3 OR (95% CI)**	**Model 1 OR (95% CI)**	**Model 2 OR (95% CI)**	**Model 3 OR (95% CI)**	**Model 1 OR (95% CI)**	**Model 2 OR (95% CI)**	**Model 3 OR (95% CI)**	**Model 1 OR (95% CI)**	**Model 2 OR (95% CI)**	**Model 3 OR (95% CI)**
Age	-	-	-	-	-	-	0.960 (0.931, 0.990)^**^	0.953 (0.922, 0.986)^**^	0.950 (0.917, 0.983)^**^	-	-	-
Female (ref: male)	0.582 (0.348, 0.973)^*^	-	-	-	-	-	-	-	-	-	-	-
Education level	-	-	-	-	-	-	-	-	-	-	0.927 (0.871, 0.987)^*^	0.897 (0.837, 0.961)^**^
Vitality (ref: 1)	-	7.791 (2.377, 25.529)^***^	4.824 (1.434, 16.231)^**^	-	2.345 (1.044, 5.268)^*^	-	-	3.106 (1.751, 5.512)^***^	2.965 (1.658, 5.301)^***^	-	2.720 (1.496, 4.948)^***^	2.455 (1.278, 4.718)^**^
Cognition (ref: 2)												
Normative cognition	-	-	-	-	-	-	-	2.339 (1.314, 4.165)^**^	2.357 (1.302, 4.268)^**^	-	-	-
Pre-MCI	-	-	-	-	-	-	-	1.941 (1.023, 3.681)^*^	**1.915 (0.997, 3.678)**	-	-	-
Psychological domain (ref: 1)	-	-	0.754 (0.649, 0.876)^***^	-	-	0.839 (0.746, 0.943)^**^	-	-	-	-	-	0.823 (0.741, 0.914)^***^
Multicomorbidity (refer 3)												
Without chronic disease	-	-	-	-	5.162 (1.877, 14.194)^***^	5.400 (1.830, 15.941)^**^	-	-	-	-	2.505 (1.124, 5.583)^*^	-
With 1 chronic disease	-	-	-	-	3.778 (1.551, 9.198)^**^	4.028 (1.549, 10.476)^**^	-	-	-	-	2.481 (1.307, 4.707)^**^	-
With 2 chronic diseases	-	-	-	-	2.867 (1.158, 7.096)^*^	3.142 (1.194, 8.270)^*^	-	-	-	-	2.143 (1.131, 4.058)^*^	-

OR, odds ratios; CI, confidence intervals; GDS, the Geriatric Depression Scale.

Model 1 is adjusted for age, gender, and education. Model 2 is adjusted for covariates in model 1 and health-related factors, including self-reported smoking, alcohol intake, chronic mutimorbidities, cognition based on normative z-scores of neuropsychological test battery. Model 3 is adjusted for covariates in model 2 and GDS.

* p < 0.05;

** p < 0.01; and

*** p < 0.001; bold values denote marginally statistical significance. Ref cognition 2 = MCI; Refer psychological domain 1 = impairment in psychological domain of IC; Ref multimorbidity 3 = with more than 2 chronic diseases. “-” = no statistical significance.

Multiple logistic regression analysis indicated that chronic disease burden had different effects on HRQoL and the physical dimension of HRQoL (Table 4). Those with less multimorbidities had a higher likelihood of better independent living and less pain. Compared to those with more than two multimorbidities, those with two multimorbidities, with one comorbidity, and those without comorbidity showed a gradually increased likelihood of better independent living in Model 2 (OR = 2.867, 95% CI = 1.158–7.096; OR = 3.778, 95% CI = 1.551; OR = 5.162, 95% CI = 1.877–14.194) and Model 3 (OR = 3.142, 95% CI = 1.194–8.270; OR =4.028, 95% CI = 1.549–10.476; OR = 5.400, 95% CI = 1.830–15.941) and less pain in Model 2 (OR = 2.143, 95% CI = 1.131–4.058; OR = 2.481, 95% CI = 1.307–4.707; OR = 2.505, 95% CI = 1.124–5.583), respectively.

### 3.4. The influence of other dimensions of IC and chronic disease burden in the psychosocial dimension of HRQoL

The capacity decline of the IC cognitive domain (no more than MCI level) did not affect the psychological dimension of HRQoL and the reserve decline in vitality and psychological domains of IC, and chronic burden had a domain-specific influence on the psychosocial dimension of HRQoL. Compared to those with low vitality, individuals with high reserve showed a significantly higher likelihood of better mental health in Model 2 (OR = 5.613, 95% CI = 1.916–16.443) and Model 3 (OR = 5.539, 95% CI = 1.625–18.880), more happiness in Model 2 (OR = 2.445, 95% CI = 1.290–4.631), and better relationships in Model 2 (OR = 3.989, 95% CI = 1.517–10.488) and Model 3 (OR = 3.467, 95% CI = 1.169–10.279). Those with lower capacity of IC psychological domain (higher GDS score) had a low likelihood of better mental health (OR = 0.705, 95% CI = 0.598–0.831), more happiness (OR = 0.784, 95% CI = 0.702–0.875), higher self-worth (OR = 0.784, 95% CI = 0.678–0.907), less coping (OR = 0.721, 95% CI = 0.587–0.886), and better relationships (OR = 0.799, 95% CI = 0.696–0.918) after being adjusted for all confounders in Model 3.

Multiple logistic regression analysis indicated that chronic disease burden had a significant influence on mental health, coping, relationships, and the psychosocial dimension of HRQoL (Table 5). Compared to those with more than two multimorbidities, individuals with two multimorbidities (OR = 1.668, 95% CI = 0.672–4.137, OR = 1.904, 95% CI = 0.479–7.566; OR = 1.845, 95% CI = 0.762–4.464), with one comorbidity (OR = 3.041, 95% CI = 1.265–7.311; OR = 4.636, 95% CI = 1.310–16.408; OR = 1.913, 95% CI = 0.799–4.580), and without comorbidity (OR = 4.561, 95% CI = 1.636–12.719; OR = 11.273, 95% CI = 2.993–42.453; OR = 4.752, 95% CI = 1.811–12.469) indicated gradually increased likelihood of better mental health, less coping, and better relationships after being adjusted for confounders in Model 2. However, no significant differences were observed between individuals with more than two multimorbidities and those with two multimorbidities. A significant difference was also not observed between individuals with more than two multimorbidities and those with one comorbidity in relationships. Those with two multimorbidities (OR = 1.459, 95% CI = 0.360–5.921; OR = 1.646, 95% CI = 0.636–4.263), with one comorbidity (OR = 2.635, 95% CI = 0.711–9.767; OR = 1.686, 95% CI = 0.659–4.315), and without comorbidity (OR = 5.907, 95% CI = 1.466–23.796; OR = 4.714, 95% CI = 1.678–13.242) indicated the gradually increased likelihood of less coping and better relationships after being adjusted for confounders in Model 3. However, the number of multicomorbidities did not have a significant influence on coping and relationships. Individuals with CVD had a significantly low likelihood of better mental health (OR = 1.925, 95% CI = 1.039–3.568) after being adjusted for confounders in Model 3. Age, education level, and smoking also indicated a significant influence on mental health.

**Table 5:** Multiple logistic regression analysis of the association of different IC domains and psychological dimension of HRQoL.

	**Mental health**			**Happiness**			**Self-worth**			**Coping**			**Relationships**		
	**Model 1 OR (95% CI)**	**Model 2 OR (95% CI)**	**Model 3 OR (95% CI)**	**Model 1 OR (95% CI)**	**Model 2 OR (95% CI)**	**Model 3 OR (95% CI)**	**Model 1 OR (95% CI)**	**Model 2 OR (95% CI)**	**Model 3 OR (95% CI)**	**Model 1 OR (95% CI)**	**Model 2 OR (95% CI)**	**Model 3 OR (95% CI)**	**Model 1 OR (95% CI)**	**Model 2 OR (95% CI)**	**Model 3 OR (95% CI)**
Age	-	1.060 (1.019, 1.103)^**^	1.062 (1.016, 1.111)^**^	-	-	-	-	-	-	0.945 (0.902, 0.990)^*^	-	-	-	-	-
Education level	-	0.912 (0.846, 0.984)^*^	0.895 (0.823, 0.974)^**^	-	-	-	-	-	-	-	-	-	-	-	-
Smoking (refer 3)															
Never smoking	-	-	0.238 (0.085, 0.669)^**^	-	-	-	-	-	-	-	-	-	-	-	-
Ever smoking	-	-	0.220 (0.050, 0.962)^*^	-	-	-	-	-	-	-	-	-	-	-	-
Vitality (refer 1)	-	5.613 (1.916, 16.443)^**^	5.539 (1.625, 18.880)^**^	-	2.445 (1.290, 4.631)^**^	-	-	-	-	-	-	-	-	3.989(1.517, 10.488)^**^	3.467 (1.169, 10.279)^*^
Psychological domain (refer 1)	-	-	0.705 (0.598, 0.831)^***^	-	-	0.784 (0.702, 0.875)^***^	-	-	0.784 (0.678, 0.907)^***^	-	-	0.721(0.587, 0.886)^**^	-	-	0.799(0.696, 0.918)^***^
Multicomorbidity (refer 3)															
Without chronic disease	-	4.561 (1.636, 12.719)^**^	-	-	-	-	-	-	-	-	11.273 (2.993, 42.453)^***^	5.907(1.466, 23.796)^*^	-	4.752 (1.811 12.469)^**^	4.714 (1.678, 13.242)^**^
With 1 chronic disease	-	3.041 (1.265, 7.311)^*^	-	-	-	-	-	-	-	-	4.636 (1.310, 16.408)^*^	2.635 (0.711, 9.767)	-	1.913 (0.799, 4.580)	1.686 (0.659, 4.315)
With 2 chronic diseases	-	1.668 (0.672, 4.137)	-	-	-	-	-	-	-	-	1.904 (0.479, 7.566)	1.459 (0.360, 5.921)	-	1.845 (0.762, 4.464)	1.646 (0.636, 4.263)

OR, odds ratios; CI, confidence intervals; ARHL, age-related hearing loss; GDS, the Geriatric Depression Scale.

Model 1 is adjusted for age, gender, and education. Model 2 is adjusted for covariates in model 1 and health-related factors, including self-reported smoking, alcohol intake, chronic multicomorbidities, and cognition based on normative z-scores of neuropsychological test battery. Model 3 is adjusted for covariates in model 2 and GDS. Ref smoking 3 = current smoking; Ref vitality 1 = capacity decline of vitality; Refer psychological domain 1 = impairment in psychological domain of IC; Ref multicomorbidity 3 = with more than 2 chronic diseases.

* p < 0.05;

** p < 0.01; and

*** p < 0.001; “-” = no statistical significance.

## 4. Discussion

In this cross-sectional study, we found that IC domains had different influences on HRQoL and domain-specific HRQoL. Individuals with sensory impairment indicated low HRQoL in senses of physical dimensions. Those with locomotion impairment had significantly low HRQoL in independent living and pain in the physical dimension. The combined effects of sensory and locomotion domains in IC indicated low HRQoL in all three domains of the physical dimension (Table 3). Participants with cognitive impairment of IC showed a significantly low HRQoL in the senses of physical dimensions. The impairment in both the vitality and psychological domains of IC showed a more profound influence on physical and psychosocial dimensions. Chronic disease burden, referred to as multimorbidities, mainly had a significant influence on independent living in the physical dimension of HRQoL. Those with more multimorbidities had a higher likelihood of worse independent living.

Low IC reserve has been verified to be associated with worse physical, mental, and total QoL (Ma et al., 2020). Some domains of IC, such as sensory (Harithasan et al., 2020; Zhang et al., 2021), locomotion (Henchoz et al., 2017), cognition (Bouldin et al., 2021), and depressive symptoms (Toyoshima et al., 2021), were shown to be related to HRQoL. The present study extends our knowledge about the different effects of IC domains on domain-specific HRQoL. First, we confirmed that the sensory and locomotion domains of IC have different effects on HRQoL, and individuals with sensory and locomotion impairments have worse HRQoL than those with sensory or locomotion impairments. Individuals with sensory impairment, referred to as hearing (probably with tinnitus) and/or vision impairment, had a significantly worse sense of physical dimension after being adjusted for demographic, medical, and psychological confounders. The result was similar to the reported results of physical dimension-specific HRQoL in previous studies (Harithasan et al., 2020; Ding et al., 2023). We define sensory impairment based on previous studies that showed that hearing loss and/or tinnitus (Zhang et al., 2021) and vision impairment (Xiang et al., 2020) had adverse effects, and dual sensory impairment (Harithasan et al., 2020; Ho et al., 2021) and multisensory impairment (Liljas et al., 2020) had worse effects on HRQoL. Although self-reported poor hearing or poor vision were not associated with reduced QoL in longitudinal follow-up, the relationships between hearing loss or vision impairment and self-rated health were significantly associated in cross-sectional analyses after adjusting for sociodemographic and lifestyle factors, chronic illness, mobility limitations, and cognition (Yu and Liljas, 2019). Since locomotion contains muscle function (Cesari et al., 2018), locomotion impairment of IC assessed by a slow gait and/or weak grip significantly influences overall HRQoL, including independent living and pain in the physical dimension. Those with low locomotion reserve had a higher likelihood of worse HRQoL and severe pain after adjustment for demographic and health-related confounders in Model 1, and less independent living after adjustment for all confounders in Model 3. A similar result had been reported that mobility had a strong impact on overall QoL after adjustment for demographic, socioeconomic, and health covariates (Henchoz et al., 2017). It was found that the locomotion domain of IC could mediate the adverse impact of the sensory domain (vision impairment) on wellbeing (Ho et al., 2021). Indeed, the combined effect of sensory and locomotion impairment on overall and physical dimension-specific HRQoL was more severe than that of sensory or locomotion impairment. Those with sensory and locomotion impairment had a higher likelihood of worse overall HRQoL and HRQoL in all domains of the physical dimension, including independent living, senses, and pain.

We did not find the effect of sensory impairment on mental health in the psychological dimension of HRQoL that was reported in the previous literature (Ding et al., 2023). A cause might result from a different HRQoL scale. In addition, we did not completely exclude the effect of the partial collinearity of the independent variable IC and the dependent variable on the results. However, sensory functions in the independent variable IC and the dependent variable HRQoL are not the same concept. First, senses in HRQoL contain hearing- and vision-related communication ability: “How well do you communicate with others (e.g., talking, signing, texting, being understood by others, and understanding them?)”. Second, when individuals with hearing loss are assessed by a PTA value of < 25 in the sensory domain of IC, these individuals often do not feel poor senses, including sensory impairment or the decline of communication ability.

Second, this study also verified that the vitality domain of IC and multimorbidity have different effects on overall HRQoL and domain-specific HRQoL. Among IC domains, vitality had a more profound effect on overall HRQoL and domain-specific HRQoL. After adjustment for all confounders, individuals with vitality decline had a significantly higher likelihood of worse overall HRQoL, HRQoL in senses and pain, independent living (in Model 2) of physical dimension, and mental health and relationships and happiness (in Model 2) in the psychological dimension. Compared with IC, chronic disease burden mainly had a profound impact on HRQoL in the physical dimension and some domains in the psychological dimension. Participants with more multimorbidities had a higher likelihood of worse independent living and pain in the physical dimension and a significantly higher likelihood of worse HRQoL in coping and relationships in the psychosocial dimension after adjusting for all confounders, and mental health after adjustment for demographic and health-related confounders in Model 2. The vitality domain reflects the energy metabolic capacity for the maintenance of an individual's optimal homeostasis level (Cesari et al., 2018). Vitality capacity decline is the basis of other IC domains (Cesari et al., 2018; Bautmans et al., 2022) and therefore has widespread effects on HRQoL. However, the operational definition of vitality capacity is absent. The assessment of vitality was different in previous studies. Some studies were based on malnutrition, such as weight loss or weight gain, abnormal BMI, and abdominal fat (Cigolle et al., 2007; Ma et al., 2020). The number of multimorbidities (Stephens et al., 2020), hand grip strength (Beard et al., 2019, 2021; Huang et al., 2021), forced expiratory volume (Lewsey et al., 2020; Beard et al., 2021), and hormonal status that reflects physiological changes (Beard et al., 2019, 2021) were also included in the vitality assessment. In the 36-Item Short Form (SF-36) health survey instrument, vitality assessment was based on four simple questions: full of life; have a lot of energy; feel tired; and feel worn out (Zhu et al., 2021). Recently, a WHO conceptual working definition was proposed that vitality capacity is a physiological state (due to normal or accelerated biological aging processes) resulting from the interaction between multiple physiological systems, reflected in (the level of) energy and metabolism, neuromuscular function, and immune and stress response functions of the body (Bautmans et al., 2022). According to the definition, some tools, such as the resting energy expenditure, submaximal energy expenditure, peak energy expenditure, exercise intolerance assessed by a 6-min walk, and peak oxygen consumption during cardiopulmonary testing (peak VO_2_), may be objective to assess vitality (Schrack et al., 2010; Lewsey et al., 2020). After an integrated vitality assessment of instruments in previous studies, we chose fatigue, physical activity, and abnormal weight as the main parameters for vitality assessment in this study. The vitality construct needs to be further validated in the longitudinal study.

In addition, among IC domains, the psychological domain was another domain that has a significant influence on overall and domain-specific HRQoL, apart from the vitality domain. Those with depressive symptoms had a higher likelihood of worse overall HRQoL, independent living, and pain in the physical dimension and all five domains in the psychosocial dimension after adjustment for all confounders in Model 3. Cognitive impairment also only significantly affected the HRQoL of the senses in the physical dimension, which was consistent with our previous study (Zhang et al., 2021). Those with poor cognitive function had a high likelihood of worse senses. The close association between cognition and sensory function has been verified (Albers et al., 2015; Wayne and Johnsrude, 2015; Jafari et al., 2019). There is a bidirectional interaction between the central auditory systems and the cognitive brain, and central auditory processing, an important component of cognition performance, is closely associated with senses in the physical dimension (Ruan et al., 2023). Aging, vascular impairment, and chronic inflammation are the common risk factors for hearing and cognitive impairment. Some minor impairments, such as cortical microinfacts, have been confirmed to be the only subtype of microvascular lesions, which is a consistent determinant of cognitive decline across the age spectrum from vascular to mixed dementia (Costanza et al., 2012). The common risk factors and pathological association support our above conclusion. Moreover, cognitive decline, a common neurological disease in elderly, characterized by biological damage in the limbic system of the brain, is considered an exemplification of how, from a neurobiological abnormality, one transitions to a psychological feeling (hopelessness, which is also one of the subcomponents of the definition of demoralization) and vice versa (Clarke and Kissane, 2002; Costanza et al., 2015). In fact, depression might be the intermediate mediator between cognitive impairment and mental health (Toyoshima et al., 2021). Unfortunately, the reported negative relationship between cognitive impairment with mental health in the psychological dimension of HRQoL (Bouldin et al., 2021) was not found in this study.

The major strengths of this study include the objective cognitive measures based on the normative *z*-scores of six neuropsychological tests and the process *z*-scores of the Hopkins Verbal Learning Test-Revised (Ruan et al., 2021b) and auditory assessment by using pure-tone audiometry. Moreover, the independent influence of IC domains on overall and domain-specific HRQoL in physical and psychological dimensions was investigated. This study has some limitations. First, chronic disease burden might include chronic disease and its severity, aside from the number of multimorbidities. CVD, physical frailty, and its severity were shown as independent risk factors that affect overall and domain-specific HRQoL in older adults with age-related hearing loss and tinnitus (Zhang et al., 2021). Another limitation of this study was that we did not analyze the effects of chronic disease and its severity on HRQoL due to the small sample size. Third, we assessed the psychological domain only based on depressive symptoms in this study, which is also a limitation. In fact, social dysfunction was shown to be an independent risk factor that affected total HRQoL and domain-specific HRQoL in age-related hearing loss with tinnitus in older adults (Zhang et al., 2021). According to the definition suggested by the WHO, the psychological domain of IC includes mood and sociality (Cesari et al., 2018). Demoralization might be a promising component of the psychological construct of IC (Clarke and Kissane, 2002; Costanza et al., 2020, 2022). In accordance with the original definition by Clarke and Kissane (Clarke and Kissane, 2002), demoralization includes many of the feelings that the elderly can experience. The demoralization applies to a broader segment of patients, for whom the criteria for depression according to the Diagnostic and Statistical Manual of Mental Disorders (DSM) are not met. It was developed precisely in the somatic context, in patients with multiple diseases (disease burden). Demoralization has also been shown to be statistically associated with suicidal ideation and suicidal behavior, even for individuals for whom all DSM V criteria cannot be completed. Because of this, demoralization often risks being underestimated and having major consequences (Costanza et al., 2022). Interpersonal relationships and interpersonal help were emphasized as having pivotal significance among the elderly (Costanza et al., 2020). Therefore, further investigation of the combined effects of depressive symptoms and social dysfunction on overall and domain-specific HRQoL was required in the future. In addition, the cross-sectional study made it impossible to define the causal relationship between IC domains and HRQoL due to the cross-sectional nature of the data. Meanwhile, we did not perform appropriate statistical corrections in the multiple regression analysis. In fact, multiple testing corrections are mainly used in clinical trials to decrease false positive results and lead to unnecessary harm. To perform multiple testing corrections in this observational study might be too conservative to cause the loss of potential risk factors with important clinical significance. Last but not least, the absence of uniform screening tools for IC domains and HRQoL might result in different conclusions. For example, the psychological domain of IC might be assessed based on depressive symptoms by using different simple questionnaires, such as the GDS15 (Zhang et al., 2021) and the Generalized Anxiety Disorder-7 scale (Huang et al., 2021), the Epidemiological Studies-Depression (CES-D) scale, and sleep disturbance (Huang et al., 2021; Zhu et al., 2021).

The heterogeneous influence of IC domains and multimorbidities on overall and domain-specific HRQoL has important clinical significance in the personal management of older adults to achieve healthy aging. The declines in overall and domain-specific HRQoL were mediated more strongly and extensively by IC than multimorbidity. IC might be a more sensitive predictive parameter for the changes in HRQoL in older populations than multimorbidity. Individuals with different frailty phenotypes due to the reserve decline of different IC domains might have different dimensions and subdomain impairments of HRQoL. For instance, sensory and locomotion domains were important components of the physical frailty phenotype. The results indicated a difference in prevention targets for individuals with IC decline and multimorbidities. Therefore, to perform the guidance for the person-centered assessment of IC (World Health Organization, 2019), integrated care for older people with a decline in IC reserve (World Health Organization, 2017b) is very necessary.

## 5. Conclusion

In conclusion, the influence of IC domains and multimorbidity on HRQoL was heterogeneous. There was a synergistic effect of sensory and locomotion domains of IC on the physical dimension of HRQoL. The reserve decline of sensory, locomotion, and cognitive domains mainly affects the physical dimension of HRQoL, with sensory and cognitive domains corresponding to senses and locomotion corresponding to independent living and pain. The reserve decline in both vitality and psychological domains had a more extensive influence on HRQoL, including physical and psychosocial dimensions. Multimorbidities showed a negative influence on independent living and pain in the physical dimension. Based on the more strong and more extensive effects of IC than multimorbidities on HRQoL, person-centered assessment of IC domains and integrated care were critical to achieving healthy aging for older people.

## Data availability statement

The original contributions presented in the study are included in the article/Supplementary material, further inquiries can be directed to the corresponding authors.

## Ethics statement

The studies involving human participants were reviewed and approved by the Ethics Committee of Huadong Hospital (Approval No. Ref 2018K055). The patients/participants provided their written informed consent to participate in this study.

## Author contributions

QR and ZY conceived and designed the manuscript. QR wrote the manuscript. JC, ZB, ZY, and QR revised the manuscript. XH, JR, JC, ZB, QR, and WZ conducted the investigation and data analysis. All authors contributed to the article and approved the submitted version.
