# Supplementary material for: The overall and domain-specific quality of life of Chinese community-dwelling older adults: the role of intrinsic capacity and disease burden
Source: Front Psychol. 2023 Aug 25;14:1190800. doi: 10.3389/fpsyg.2023.1190800 (PMC10485271; doi:10.3389/fpsyg.2023.1190800)
Supplement: Supplementary file 1 [file Table_1.docx]

Supplementary table 1. Characteristics of Study Participants

|  | Total sample | Lack of sensory and locomotion impairment (n=40) | Sensory impairment (n=209) | Locomotion impairment (n=15) | Sensory and locomotion impairment (n=164) | F/X²value | p |
| --- | --- | --- | --- | --- | --- | --- | --- |
| Age | 429 | 69.08±5.640 | 71.42±6.729 | 76.00±6.969 | 75.39±6.725 | 17.69 | <0.0001 |
| Female | 429 (246) | 40 (19) | 209 (126) | 15 (4) | 164 (96) | 8.1843 | 0.0707 |
| Education | 427 |  |  |  |  | 69.9854 | 0.0886 |
| BMI | 421 | 24.95±2.999 | 23.79±2.899 | 24.45±3.224 | 24.61±3.844 | 2.505 | 0.0587 |
| Somking | 422 | 40 | 202 | 15 | 164 | 15.446 | 0.0171 |
| Never smokers | 353 | 27 | 168 | 14 | 143 |  |  |
| Ever smokers | 38 | 8 | 14 | 1 | 15 |  |  |
| Current smokers | 31 | 5 | 20 | 0 | 6 |  |  |
| Drinking | 422 | 40 | 202 | 15 | 164 | 19.226 | 0.0038 |
| Never drinkers | 378 | 29 | 184 | 13 | 151 |  |  |
| Ever drinkers | 14 | 3 | 9 | 0 | 2 |  |  |
| Current drinkers | 30 | 8 | 9 | 2 | 11 |  |  |
| Multicomorbidity | 398 |  |  |  |  | 24.219 | 0.0040 |
| without chronic disease | 49 | 4 | 28 | 3 | 14 |  |  |
| with 1 chronic disease | 127 | 16 | 69 | 3 | 39 |  |  |
| with 2 chronic diseases | 124 | 10 | 55 | 8 | 50 |  |  |
| With >2 chronic diseases | 98 | 6 | 36 | 1 | 55 |  |  |
| Vitality |  |  |  |  |  | 16.913 | 0.0007 |
| no decline | 314 | 37 | 161 | 11 | 105 |  |  |
| decline | 108 | 4 | 43 | 3 | 59 |  |  |
| Cognition | 427 |  |  |  |  | 24.026 | 0.0005 |
| Normal | 215 | 30 | 112 | 8 | 65 |  |  |
| Pre-MCI | 111 | 8 | 54 | 5 | 44 |  |  |
| MCI | 103 | 2 | 43 | 2 | 55 |  |  |
| GDS score | 420 |  |  |  |  | 52.7945 | 0.1983 |
| ≤4 | 289 | 27 | 147 | 12 | 103 |  |  |
| ≥5 | 131 | 11 | 61 | 2 | 57 |  |  |

Pre-MCI, pre-mild cognitive impairment; MCI, mild cognitive impairment; GDS, the Geriatric Depression Scale; *p < 0.05; **p < 0.01; and ***p < 0.001.
